# Hpa1 is a type III translocator in *Xanthomonas oryzae* pv. *oryzae*

**DOI:** 10.1186/s12866-018-1251-3

**Published:** 2018-09-04

**Authors:** Xuan Wang, Liyuan Zhang, Hongtao Ji, Xuyan Mo, Ping Li, Junzhi Wang, Hansong Dong

**Affiliations:** 1 Department of Plant Pathology, College of Plant Protection, Nanjing Agricultural University, Nanjing, 210095 Jiangsu Province China; 2 Present address: Department of Biology, College of Life Sciences, Jiangsu Formal University, Xuzhou, 221116 Jiangsu Province China

## Abstract

**Background:**

Pathogenic Gram-negative bacteria interact with their eukaryotic hosts by deploying the type III translocon to inject effector proteins into the cytosol of eukaryotic cells. The translocon compositions, the number and biochemical characteristics of type III translocators in animal-pathogenic bacteria have been well elucidated, but information is lacking for plant-pathogenic bacteria. With extensive studies on biological functions of the Hpa1 protein secreted by the type III secretion system in *Xanthomonas oryzae* pv. *oryzae* (*Xoo*), we show here that Hpa1 is a type III translocator based on measurements of two proteins categorized as transcription activator-like (TAL) effector.

**Results:**

Hpa1 was functionally associated with the TAL effector PthXo1 or AvrXa10 by genetic analysis of the wild-type *Xoo* strain and related mutants or recombinant strains. Inoculation experiments suggested that Hpa1 is required not only for the virulent role of PthXo1 in the susceptible rice variety Nipponbare, but also for the avirulent function of AvrXa10 on the resistant rice variety IRBB10. Hpa1 is unrelated to the secretion of PthXo1 and AvrXa10 out of bacterial cells. However, Hpa1 is critical for both TAL effectors to be translocated from bacterial cells into the cytosol of rice cells based on replicate experiments performed on the susceptible and resistant varieties, respectively. Hpa1-mediated translocation of PthXo1 is coincident with induced expression of rice *SWEET11* gene, which is the regulatory target of PthXo1, resulting in the occurrence of the bacterial blight disease in the susceptible rice variety. By contrast, the immune hypersensitive response is induced in agreement with induced expression of rice *Xa10* gene, which is the target of AvrXa10, only when AvrXa10 is translocated from bacteria into cells of the resistant rice variety. All the virulent or avirulent performances of the TAL effectors are nullified by directed mutation that removes the α-helix motif from the Hpa1 sequence.

**Conclusions:**

The genetic and biochemical data demonstrate that Hap1 is a type III translocator at least for TAL effectors PthXo1 and AvrXa10. The effect of the directed mutation suggests that Hpa1 depends on its α-helical motif to fulfil the translocator function.

**Electronic supplementary material:**

The online version of this article (10.1186/s12866-018-1251-3) contains supplementary material, which is available to authorized users.

## Background

Many effector proteins of Gram-negative bacteria, which are plant [1] or animal [2, 3] pathogens, are secreted by the type III secretion system (T3SS). Subsequently, effectors must be translocated into the cytosol of eukaryotic cells in order to fulfill virulent or avirulent roles [1, 4]. Effector translocation relies on the type III translocon, which is made of hydrophilic and hydrophobic proteinaceous translocators [2, 3, 5]. The distinct translocators make up the translocon by complicated monogenous and heterogeneous molecular interactions and by associations with recognition compounds at the plasma membranes (PMs) of eukaryotic cells [2, 3]. In the current model [2, 5], hydrophilic translocators form complexes at the tip [6, 7] of proteinaceous pili in plant pathogens or proteinaceous needles in animal pathogens and associate with hydrophobic counterparts, which oligomerize into the target PM [8, 9]. The resulting translocon consists of an inner conduit that is connected to a pilus or needle channel and opens into eukaryotic cells, thereby allowing the direct injection of effectors into the cytosol of eukaryotic cells [2, 3, 9].

All hydrophilic translocators so far identified in plant-pathogenic bacteria are characteristic of harpins, which belong to T3SS accessory proteins and contain a unitary hydrophilic domain or an additional enzymatic domain [5, 8–12]. While two-domain harpins may target the bacterial periplasm or plant cell walls to facilitate pilus growth [8, 10, 11], one-domain harpins localize to plant PMs, with a potential role in the translocon assembly [8, 9, 13, 14]. Eukaryotic PM sensors, which are either lipids [2, 3, 15] or proteins [13, 14], are assumed to be indispensable for translocator recognition and translocon formation [2, 3, 5, 6].

We have extensively studied multiple functions of Hpa1 [5, 14, 16], a one-domain harpin [17] from *Xanthomonas oryzae* pv. *oryzae* (*Xoo*). *Xoo* causes bacterial blight in rice (*Oryza sativa*) through effector proteins, either transcription activator-like (TAL) effectors or non-TAL effectors like Xops, *Xanthomonas* outer proteins [4, 18]. TAL effectors were found initially in plant-pathogenic bacteria of the *Xanthomonas* genus [19] and proved then to include previously designated Avr proteins present in the same pathogens [20]. TAL and non-TAL effectors are secreted along with harpins by the T3SS in a chronological pattern [2, 21] and then translocated into plant cells to play a virulent or avirulent role depending on plant varieties [1, 4]. Like the movement of all bacterial effectors, TAL and non-TAL protein translocation is a moment travel through a translocon hypothetically made of translocators [2, 22], which include one-domain harpins, such as Hpa1, in plant pathogenic bacteria [5, 8, 9, 12]. However, the role of Hpa1 in effector translocation has not been demonstrated.

Hpa1 comprises 139 amino acids with a molecular mass of 15 kD and carries a pair of α-helical coiled-coil motif at sites 37–52 and 87–102 [23]. While the N-terminal α-helix governs Hpa1 oligomerization [23], the first 53 residues determine its bioactivities in plants [5, 14, 24, 25]. Hpa1 affects virulence of the *Xoo* strain PXO99 in susceptible *japonica* rice variety Nipponbare [5] while the virulence is provided by the TAL effector PthXo1 [26]. By contrast, the TAL effector AvrXa10 of the *Xoo* strain PXO86 plays an avirulent role in resistant *indica* rice variety IRBB10 [27]. While AvrXa10 activates the host resistance gene *Xa10* to confer the immune or hypersensitive response (HR) in IRBB10 [27], PthXo1 induces virulence by activating the host susceptibility gene *SWEET11* (synonym *Os8N3*) in Nipponbare [26]. We report here that Hpa1 is a type III translocator and executes this function through the α-helical motifs.

## Methods

### Plant growth

Seeds of Nipponbare were initially provided by our colleague Professor Hongsheng Zhang (College of Agronomy, Nanjing Agricultural University) and reproduced and maintained in this lab. Rice seeds were germinated in flat plastic trays filled with a substrate containing peat, sand, and vermiculite (1:1:1 *v*/v). Three days later, the germinal seedlings were moved into 12-L pots (2 plants per pot) filled with soil from a local rice grower field. Seeds were incubated and the plants were grown in environment-controlled chambers under 28 °C, 12-h light at 250 ± 50 μmol quanta/m^2^/sec, and a relative humidity of 85%. Tobacco plants were grown in a greenhouse for two months prior to use.

### Bacterial strains and molecular vectors

Bacterial strains and plasmid vectors used and created in the present study and information on antibiotic resistance are listed in Additional file 1: Table S1. *Escherichia coli* was grown at 37 °C in Luria-Bertani broth (LB) or on LB agar (LA) plates with the appropriate antibiotics. *Xoo* strains were cultured at 28 °C on nutrient broth (NB) or NA agar (NA) medium [28]. Bacteria were cultured on medium supplemented with 100 μg/mL ampicillin, 100 μg/mL spectinomycin, or 50 μg/mL kanamycin.

### Bacterial gene modifications

The *hpa1* and *pthXo1* genes were deleted from PXO99 by using the unmarked deletion method [23]. Upstream and downstream flanking partial sequence fragments of *hpa1* or *pthXo1* were amplified from the PXO99 genomic DNA and connected together by overlapped fusion-PCR using specific primers (Additional file 1: Table S2). Every PCR product was confirmed by sequencing and then cloned into the vector pK18*sacB* by digestion with *Bam*HI and *Xba*I and ligation with T4 ligase (Thermo Scientific). Every recombinant vector was introduced into PXO99 cells by electroporation, followed by single-colony selection on kanamycin-containing and sugar-absent NA plates. Colonies from single crossovers were transferred to NB broth, grown at 28 °C for 12 h and then transferred onto plates containing NA and 10% sucrose. Sucrose-resistant colonies were replica streaked onto NA plates with and without kanamycin supplementation. Colonies resulting from double crossover events were selected based on kanamycin-negative and sucrose-positive traits, and unmarked mutants were confirmed by PCR amplification of *hpa1* and *pthXo1*, respectively. To create double mutants, pK18*sacB*:∆*hpa1* was transformed into the ∆*pthXo1* mutant.

Different tags were attached to the 3′-terminus of *pthXo1* or *avrXa10* in the pZW*pthXo1* and pZW*avrXa10* plasmid vectors. To create a *cya*-fused gene, a 1,218-bp *cya* fragment encoding amino acids 2–406 of the Cya protein was amplified from plasmid pMS107 and prefixed with the last 51-bp region of *pthXo1* that contained a *Sac*I recognition site. The recombinant sequence was inserted into pZW*pthXo1* at the *Sac*I site. A similar method was used for the construction of *avrXa10-cya*. To construct *pthXo1-blaM*, *blaM* was amplified from plasmid pBR322 using specific primers that contained a *Sal*I site. The confirmed PCR product was inserted into the *pthXo1* sequence at the *Sal*I site in pZW*pthXo1*, and *avrXa10-blaM* was similarly constructed [29]. Every recombinant vector was linearized with *Hind*III and cloned into the pHM1 vector for genetic complementation. The *hpa1pthXo1* and *hpa1avrXa10* double complementary vectors were constructed using two steps. First, the *hpa1* sequence that was linked its own promoter was cloned into pHM1 between the *Pst*I and *Kpn*I sites. Second, pZW*avrXa10 or* pZW*pthXo1* was linearized using *Hind*III and inserted into the *Hind*III site of pHM1*hpa1*. Complementation or transformation was performed by electroporation.

### Bacterial virulence/avirulence evaluation

Overnight *Xoo* NA cultures were washed twice and resuspended in sterile water to generate inoculum suspensions with an optical density of OD_600_ = 0.5. A bacterial suspension was inoculated on leaves of 2-week-old rice seedlings by infiltration with needleless syringes at 3 sites per leaf. Alternatively, a bacterial suspension was inoculated on leaves of 2-month-old adult rice plants by the leaf clipping method [28]. Symptoms were scored by photographing or measuring lesion lengths or HR areas. Bacterial growth in rice leaves was measured by harvesting 10 leaves for each treatment after needleless syringes inoculation.

### Protein secretion and translocation assays

TAL effector-related PXO99 strains were grown in NB broth at 28 °C with the appropriate antibiotics to logarithmic phase. Bacterial cells were harvested by centrifugation. The precipitated bacterial pellet was washed twice with sterile water and resuspended in 100 mL of type III-inducing XOM2 liquid media [30] to an of OD_600_ = 0.6. This XOM2 suspension was supplied with the appropriate antibiotics and incubated in a 28 °C shaker at 220 *rpm* for 16 h. XOM2 cultures were then separated into cell pellet and supernatant fractions by centrifugation. The proteins in the pellet and supernatant were extracted by sonication and by precipitation with 12.5% trichloroacetic acid, respectively [28]. Proteins were separated by 6% SDS-PAGE and transferred to Immobilon-P membranes (Millipore) for immunoblotting analyses using a Cya antibody (Santa Cruz) or β-lactamase antibody (Abcam). The ampicillin resistance protein β-lactamase protein is encoded by the pZW*tal-cya* vector (Additional file 1: Table S1), remains cell-bound unless non-specific cell leakage occurred, and was used as a control for nonspecific cell lysis. Protein blots were incubated with the specific antibody and hybridized to horseradish peroxidase-conjugated goat antimouse immunoglobulin G from the BeyoECL Plus kit (Beyotime).

The Cya reporter assay was performed on two-week-old rice seedlings inoculated with Cya-related *Xoo* strains. Bacterial suspensions were prepared from NA cultures and adjusted to an OD600 = 0.5. Each suspension was infiltrated into intercellular spaces of expanded leaves at three sites per leaf. At 12 hpi, 5-cm-long leaf segments that covered infiltration sites were excised from inoculated leaves, frozen in liquid nitrogen in a mortar, and ground with a ceramic pestle to a fine powder. The leaf powder was suspended in 350 μL of 0.1 M HCl, followed by brief centrifugation [29]. The supernatant was analyzed with a cAMP ELISA detection kit (GenScript) to determine intracellular cAMP concentrations. Total proteins in each sample for normalization were quantified by using a BCA protein assay kit (TransGen Biotech).

### Gene expression analysis

Total RNA was isolated from leaves or protoplasts by using TRIzol (Invitrogen) and treated with DNase I (Invitrogen) to remove DNA. cDNA was synthesized from RNA by using a PrimeScript RT Master Mix (TaKaRa). Quantitative real-time PCR was performed in an ABI7500 Real-Time PCR system (Applied Biosystems) using specific primers (Additional file 1: Table S2) and a SYBR Premix Ex Taq kit (TaKaRa). The constitutively expressed rice *Ubi1* gene was used as reference. The average expression level of a tested gene was quantified as transcript amount ratio to the reference gene.

### Bacterial protein preparation

To prepare proteins used in plant and protoplast treatments, *hpa1, hpa1*∆*N36, hpa1*∆*Nα, hpa1*∆*Cα*, and *hpa1*∆*NCα* genes were cloned separately into a pET30a + vector (carrying *His*) or pET41a + (carrying *GST-His)* by using *Bam*HI and *Hin*dIII. Fusion proteins were produced in BL21 under induction by IPTG according to the pET System Manual. Proteins were purified using *ProteinIso* Ni-NTA Resin (TransGen Biotech). Before use in plant and protoplast treatments, the Hpa1-His fusion protein was treated with an enterokinase (New England Biolabs**)** to remove the His tag. Purified proteins were prepared as aqueous solution stocks and their concentrations were determined using the BCA protein assay kit (TransGen Biotech). Every protein was used at a final concentration of 10 μg/mL in water [14].

### Data treatment

All experiments were carried out at least three times with similar results. Quantitative data were analyzed with the IBM SPSS19.0 software package (IBM Corporation, Armonk, NY, USA; http://www-01.ibm.com/software/analytics/spss/) according to instructions in a text book that describes in details analysis methods using IBM SPSS19.0. [31]. Homogeneity-of-variance in data was determined by Levene test, and formal distribution pattern of the data was confirmed by Kolmogorov-Smirnov test and P-P Plots [31, 32]. Then, data were analyzed by analysis of variance and least significant difference test [33–35].

## Results

### Hpa1 contributes to the virulent role of PthXo1 on rice variety Nipponbare

**Fig. 1:**
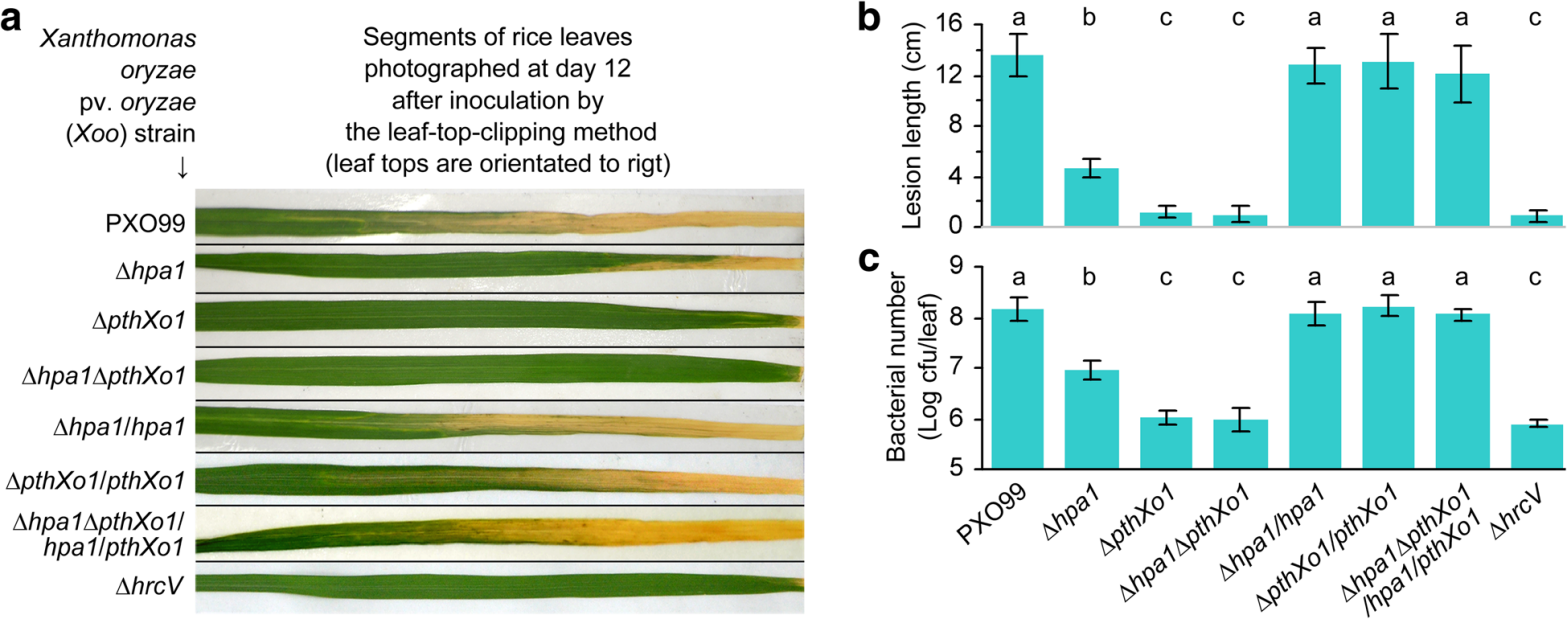
Deletion of *hpa1* and *pthXo*1 genes reduces virulence of *Xoo* strain PXO99 on the susceptible rice variety Nipponbare. Deletion of *hpa1* and *pthXo*1 genes reduces virulence of *Xoo* strain PXO99 on the susceptible rice variety Nipponbare. **a** Bacterial blight symptoms on leaves photographed at day 12 after leaf-top-clipping inoculations. **b** Blight lesion length on leaves from (a**). c** Bacterial populations shown as colony formation unit (cfu) of cultures of bacteria recovered from leaves 3 days after leaf-center-infiltrating inoculations. In (**b**) and (**c**), quantitative data are given as mean values ± statistical estimates of standard error of mean (SEM). Different letters on bar graphs indicate significant differences in multiple comparisons of data from the different bacterial strains; *P* < 0.05; *n* = 30 leaves from 3 independent experiments each involving 10 leaves in B; *n* = 9 repetitions from 3 independent experiments each involving 3 repetitions in (**c**)

**Fig. 2:**
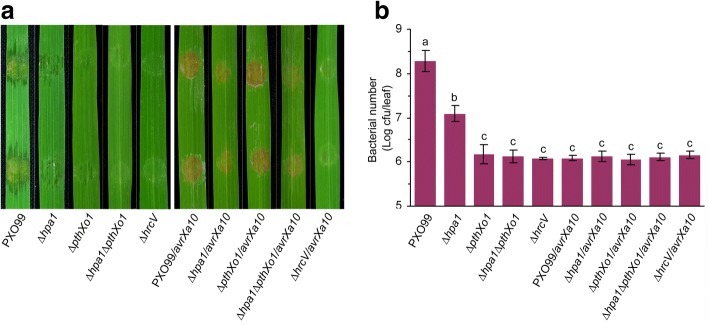
Virulence of *Xoo* TAL effector-relevant strains on IRBB10. (**a**) Virulence of PXO99 WT and TAL effector-relevant mutants on IRBB10. Fourteen-day-old IRBB10 seedlings were inoculated by leaf infiltration with every bacterial suspension of the indicated *Xoo* strains. Inoculated leaves were photographed at 5 days post-inoculation (dpi). (**b**) Bacterial populations propagated in leaf tissues were determined at 5 dpi. Quantitative data shown are means ± statistical estimates of standard error of mean (SEM); different letters on bar graphs indicated significant difference in Duncan’s multiple range tests; *P* < 0.05; number of repetition (*n*) = 9 from 3 independent experiments each involving 3 repetitions

In order to analyze the functional relationship between Hpa1 and PthXo1, we generated PXO99 mutants *∆hpa1*, *∆pthXo1*, and *∆hpa1∆pthXo1* by sequence deletion and generated *∆hpa1*/*hpa1*, *∆pthXo1*/*pthXo1*, and *∆hpa1∆pthXo1*/*hpa1*/*pthXo1* strains by genetic complementation (Fig. 1). In leaf-top-clipping (Fig. 1a) and leaf-center-infiltrating (Fig. 2a) inoculation experiments performed on the susceptible rice variety Nipponbare, these bacteria displayed extensive variations in virulence levels. Virulence degrees were quantified as the length of blight lesion on leaves observed at 12 days post-inoculation (dpi) (Fig. 1b) and bacterial populations propagated in leaf tissues 3 dpi (Fig. 1c; Fig. 2b). Bacterial populations were given as logarithmic values of colony formation units (cfu) of bacterial cells after recovery from leaf tissues. Compared to the wild-type (WT) strain, the mutants displayed highly impaired virulence based on blight lesion length and *in planta* bacterial populations. Deleting *hpa1*, *pthXo1*, or both caused substantial reductions of *in planta* bacterial populations and blight lesion length, whereas complementation strains resembled WT in terms of virulence performance (Figs. 1 and 2). In comparison, deleting *hpa1* was much less inhibitive to PXO99 virulence than *pthXo1* knockout, which almost completely nullified the bacterial virulence performance (Fig. 1a–c). This result confirms the previous finding that PthXo1 is a major determinant of the bacterial virulence [26] while Hpa1 plays a partial role in the virulence [17, 36]. Moreover, the abilities of PXO99 to cause bacterial blight (Fig. 1a, b) and to multiply in leaf tissues (Fig. 1c) were significantly (*p* < 0.01) compromised by knockout of the *hrcV* gene, which encodes an inner membrane protein essential for substrate docking into the T3SS [37]. Thus, PthXo1 requires T3SS to execute its virulence function.

### Hpa1 is required for the avirulent function of AvrXa10 on rice variety IRBB10

While PXO99 was virulent, PXO99/*avrXa10*, which was generated by introducing *avrXa10* into PXO99 cells, exhibited avirulence and induced the HR in leaves of the resistant rice variety IRBB10 (Fig. 2a). The HR appeared as brown necrotic lesion while necrosis became evident within 24 h post-inoculation (hpi) and was usually documented by leaf photography. Compared to the recombinant bacterial strain PXO99/*avrXa10* carrying a functional *hpa1* gene, the mutant *∆hpa1*/*avrXa10* generated by deleting *hpa1* was highly impaired in the HR induction level (Fig. 2a). The impaired level of HR induction was coincident with a significant (*P* < 0.01) decrease in bacterial population of *∆hpa1*/*avrXa10* propagated during the period of HR development (Fig. 2b). These data suggest the possibility that the *∆hpa1*/*avrXa10* strain incurs a partial loss of virulence due to *hpa1* deletion and the avirulent function of AvrXa10. When the *avrXa10* gene was introduced into the *hpa1*-containing *pthXo1*-knockout mutant *∆pthXo1*, resulting bacterial strain *∆pthXo1/avrXa10* performed like PXO99*/avrXa10* in the HR elicitation (Fig. 2a) and bacterial propagation (Fig. 2b). By contrast, both the HR induction and bacterial population were compromised by removing *hpa1* from the *∆pthXo1/avrXa10* genome (Fig. 2), confirming the critical effect of Hpa1 on the avirulent role of AvrX10. Moreover, the bacterial abilities to induce the HR (Fig. 2a) and to multiply in leaf tissues (Fig. 2b) were nullified by knockout of *hrcV*, suggesting that AvrXa10 requires T3SS to execute the subsequent function.

### Hpa1 does not affect secretion of PthXo1 and AvrXa10

**Fig. 3:**
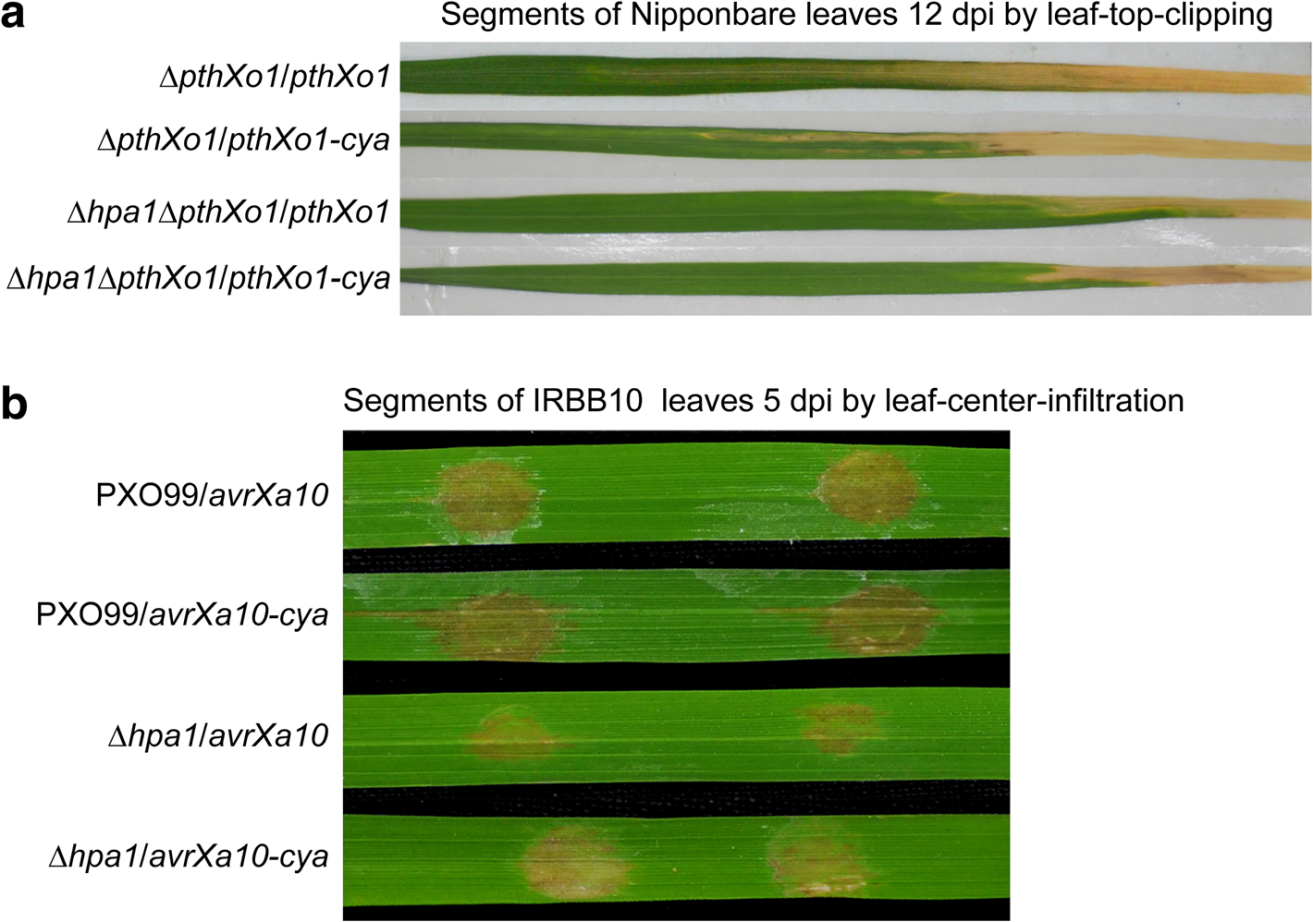
The Cya tag does not affect the virulent role of PthXo1 (**a**) and the avirulent role of AvrXa10 (**b**). Leaves were photographed at 12 dpi in (**a**) and 5 dpi in (**b**)

**Fig. 4:**
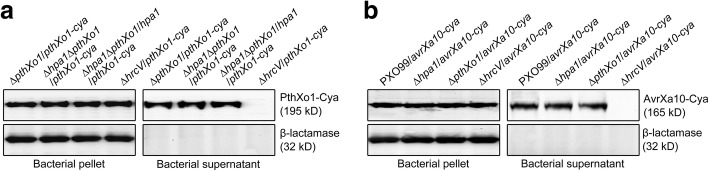
Bacterial PthXo1 (**a**) and AvrXa10 (**b**) secretion assays. Two proteins had been fused to a cya sequence and were prepared as supernatants from bacterial cultures. Protein samples were analyzed by immunoblotting with the specific antibody against Cya and the specific antibody against the β-lactamase protein used as a lysis control

We analyzed protein secretion and translocation by using calmodulin-dependent adenylate cyclase (Cya), a eukaryotic cytoplasmic import marker [8]. We verified that Cya did not affect the virulent role of PthXo1 in Nipponbare plants inoculated by the leaf-top-clipping method (Fig. 3a) and also did not affect the avirulent activity of AvrXa10 in IRBB10 plants inoculated by the leaf-center-infiltrating method (Fig. 3b). Immunoblotting analysis revealed that PthXo1-Cya was secreted by T3SS in the presence of HrcV (Fig. 4a), an *Xoo* inner membrane protein essential for substrate docking into the T3SS [28, 37]. In contrast to PthXo1, β-lactamase used as a lysis control was not secreted, instead, it remained inside bacterial cells no matter whether *hpa1* and *hrcV* were present or absent (Fig. 4a), confirming the specificity in PthXo1 secretion by the T3SS. PthXo1-Cya was secreted equally well whether or not Hpa1 was deleted, suggesting that Hpa1 was not required for TAL effector secretion. Similarly, immunoblotting analysis indicated that AvrXa10 was secreted in a HrcV-dependent manner (Fig. 2b). By contrast, β-lactamase was not secreted irrespectively of the presence or absence of *hpa1* and *hrcV* (Fig. 2b), confirming the specificity in AvrXa10 secretion by the T3SS. Hpa1 was dispensable for secretion of AvrXa10 since secretion amounts of AvrXa10-Cya were equivalent in the presence and absence of Hpa1 (Fig. 2b).

### Hpa1 is a translocator for PthXo1 and AvrXa10

**Fig. 5:**
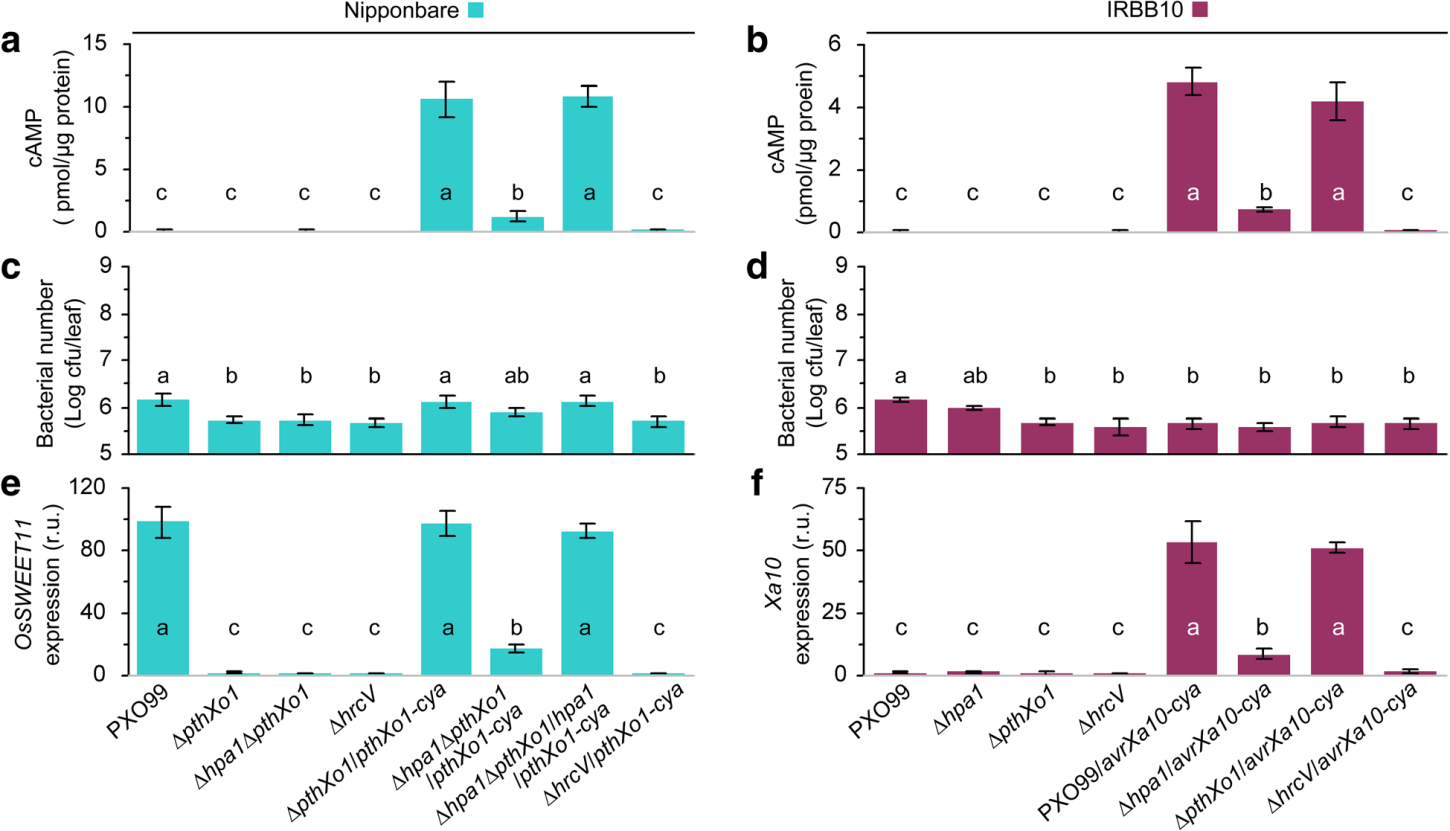
Hpa1 is a type III translocator determined to be required for translocation of PthXo1 and AvrXa21 as well as for their target gene expression. (**a**) and (**b**) Bacterial effector translocation levels shown as cAMP concentrations in leaves of Nipponbare or IRBB10 plants 12 hpi with *Xoo* strains, which are related to *hpa1*, *pthXo1*, or the secretion determinant gene *hrcV*. (**c**) and (**d**) Bacterial populations in leaves of plants 12 hpi with the indicated bacterial strains. Quantitative data are given as the means ± SEMs. (**e**) Rice *OsSWEET11* gene expression in leaves of plants equivalent to those in (**a**). The average level of gene expression in plants inoculated with the *hrcV* mutant was defined as 1 to assess relative extents of gene expression in other plants. (**f**) Rice *Xa10* gene expression in leaves of plants equivalent to those in (**b**). The average level of gene expression in plants inoculated with the *hrcV* mutant was defined as 1 to assess relative extents of gene expression in other plants. On bar graphs in (**a**) to (**f**), different letters indicate significant differences of multiple comparisons of data from the different bacterial strains; *P* < 0.05; number of repetition (*n*) = 9 from 3 independent experiments each involving 3 repetitions

In the Cya reporter assay using rice leaves inoculated by the leaf-center-infiltrating method, Hpa1 was found to be critical for PthXo1 translocation from PXO99 cells into the cytosol of Nipponbare cells. In the assay, leaves were sampled at 12 hpi, and TAL effector translocation was quantified based on cAMP concentrations during effector-Cya activity in leaf cells (Fig. 5a). High concentrations of cAMP were detected in leaves inoculated with Δ*pthXo1/pthXo1-cya* and Δ*hpa1*Δ*pthXo1*/*hpa1*/*pthXo1-cya* strains, respectively. Cya activity was substantially decreased due to *hpa1* deletion, as evidenced by a significant (*P* < 0.01) reduction in the cAMP content in leaves inoculated with the Δ*hpa1*Δ*pthXo1*/*pthXo1-cya* strain (Fig. 5a).

Hpa1 was also indispensable for AvrXa10 translocation from PXO99/*avrXa10-cya* cells in the cytosol of IRBB10 cells. The effector translocation expressed as cAMP production by AvrXa10-Cya had higher quantities with *hpa1*-containing PXO99 strains compared to *hpa1*-deleted ones (Fig. 5b). By contrast, the quantity of AvrXa10-Cya translocation was highly decreased by *hpa1* deletion, as indicated by a significant (*P* < 0.01) reduction in the cAMP content in leaves inoculated with the Δ*hpa1*/*avrXa10-cya* strain (Fig. 5b).

In the measurements of both PthXo1 (Fig. 5a) and AvrXa10 (Fig. 5b), little translocations were found with Δ*hrcV* itself, Δ*hrcV/pthXo1-cya*, or Δ*hrcV/avrXa10-cya* due to the absence of HrcV-mediated secretion (Fig. 4). Furthermore, similar amounts of AvrXa10-Cya translocation were detected in IRBB10 leaves inoculation with recombinant bacterial strains PXO99/*avrXa10-cya* and Δ*pthXo1/avrXa10-cya*, respectively (Fig. 5b). This indicates that the presence of PthXo1 does not influence the translocation of AvrXa10 from bacterial cells into the cytosol of IRBB10 cells.

In all experiments, bacterial populations in leaves had little effect on changes in TAL effector translocation within 12 hpi. At this time point, cAMP concentrations were measured to indirectly quantify translocated TAL effectors while *in planta* bacterial populations of different strains were almost similar regardless of *hpa1* deletion (Fig. 5b). Indeed, the biggest difference of the *in planta* populations between different strains was less than 3 times. In Nipponbare, ∆*hpa1*∆*pthXo1*/*pthXo1-cya* and ∆*pthXo1*/*pthXo1-cya* showed logarithmic growth rates of 5.90 and 6.15 cfu/leaf (Fig. 5b), which were converted to a 1.7-fold difference in actual bacterial populations. However, an 8.7-fold decrease in cAMP content was observed in Nipponbare leaves after inoculation with *hpa1*-deleted bacteria (Fig. 5a). In IRBB10, moreover, PXO99/*avrXa10-cya* and PXO99*∆hpa1*/*avrXa10-cya* populations had similar growth rates, approximately logarithmic 5.7 cfu/leaf (Fig. 5d), but leaf inoculation with *hpa1*-deleted bacterial cells resulted in a 6.3-fold reduction in cAMP content (Fig. 5b). Clearly, the quantitative changes in PthXo1 and AvrXa10 translocation were attributable to *hpa1* deletion, rather than differences in bacterial populations within leaf tissues, thereby indicating that Hpa1 indeed is a translocator of PthXo1 and AvrXa10.

### Hpa1-mediated translocation of PthXo1 and AvrXa10 leads to expression of the effector target genes in rice cells

To elucidate the subsequent effect of Hpa1-mediated translocation of PthXo1 on its virulent role executed via activating expression of the target gene *SWEET11* [26], we determined *SWEET11* transcript quantities in Nipponbare leaves following inoculation with proper *Xoo* strains (Fig. 5e). The analysis by RT-qPCR, namely real-time reverse transcriptase (RT) polymerase chain reaction (PCR), showed that *SWEET11* was highly expressed in plants inoculated with the WT *Xoo* strain or recombinant strains containing functional *pthXo1* and *hpa1* genes. RT-qPCR data also indicated that *SWEET11* expression was significantly (*P* < 0.01) decreased in Nipponbare leaves inoculated with bacteria in which *hpa1* or *pthXo1* or both was deleted.

RT-qPCR was also carried out to analyze the subsequent effect of Hpa1-mediated translocation of AvrXa10 on its function in activating expression of the target gene *Xa10* [27] in leaves of IRBB10 seedlings inoculated with pertinent *Xoo* strains (Fig. 5f). Strong expression of Xa10 was induced by the recombinant *Xoo* strain that has an introduced *avrXa10* gene, along with a functional *hpa1* sequence. In comparison, Xa10 expression level incurred significant (*P* < 0.01) reductions when *hpa1* or *avrXa10* or both was absent in inoculated IRBB10 plants.

### The α-helical motif is required for the function of Hpa1 as a T3SS translocator

**Fig. 6:**
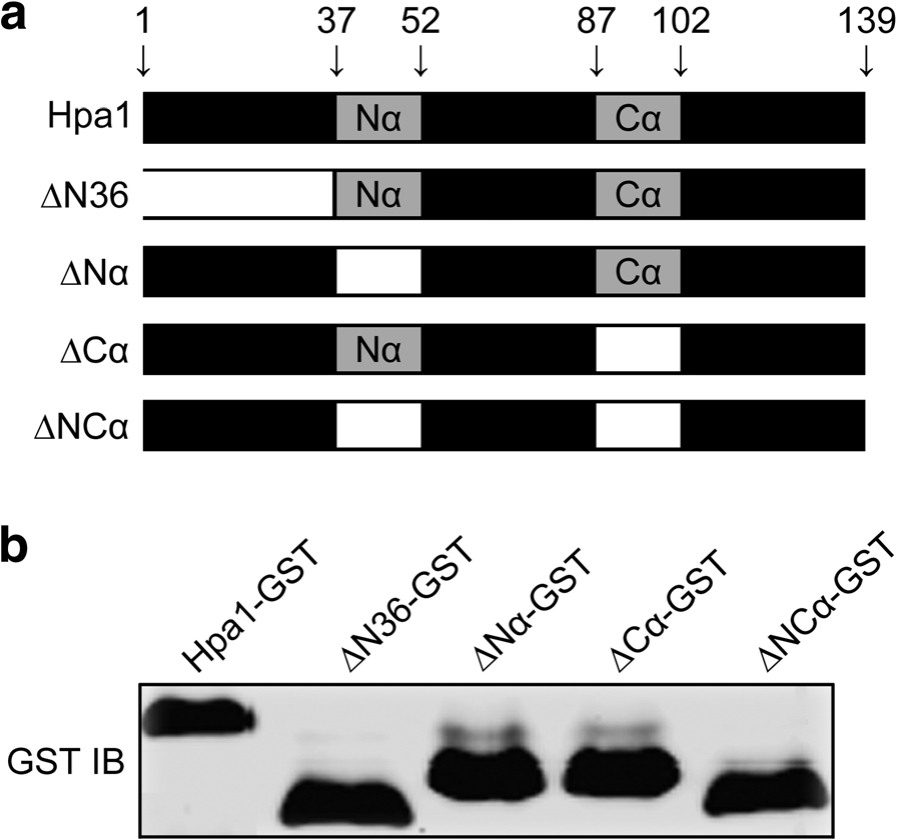
Generation and immunoblotting of Hpa1 mutant versions compared with the canonical form. **a** Schematic diagrams showing full-length sequence and mutant versions of the Hpa1 protein. Mutants ∆N36, ∆Nα, ∆Cα, and αNC were generated by deleting the N-terminal region made of 36 residues, the N-terminal helix (Nα), the C-terminal helix (Cα), and both helices (NCα), respectively. Amino acid sequence borders of these protein variants are pointed by arrowheads linked to the numbers of initial and terminal residue sites. **b** Immunoblotting. Each of Hpa1 variants was fused to a GST tag. Fusion proteins were produced by prokaryotic expression and analyzed by Western blotting hybridization with the GST antibody

**Fig. 7:**
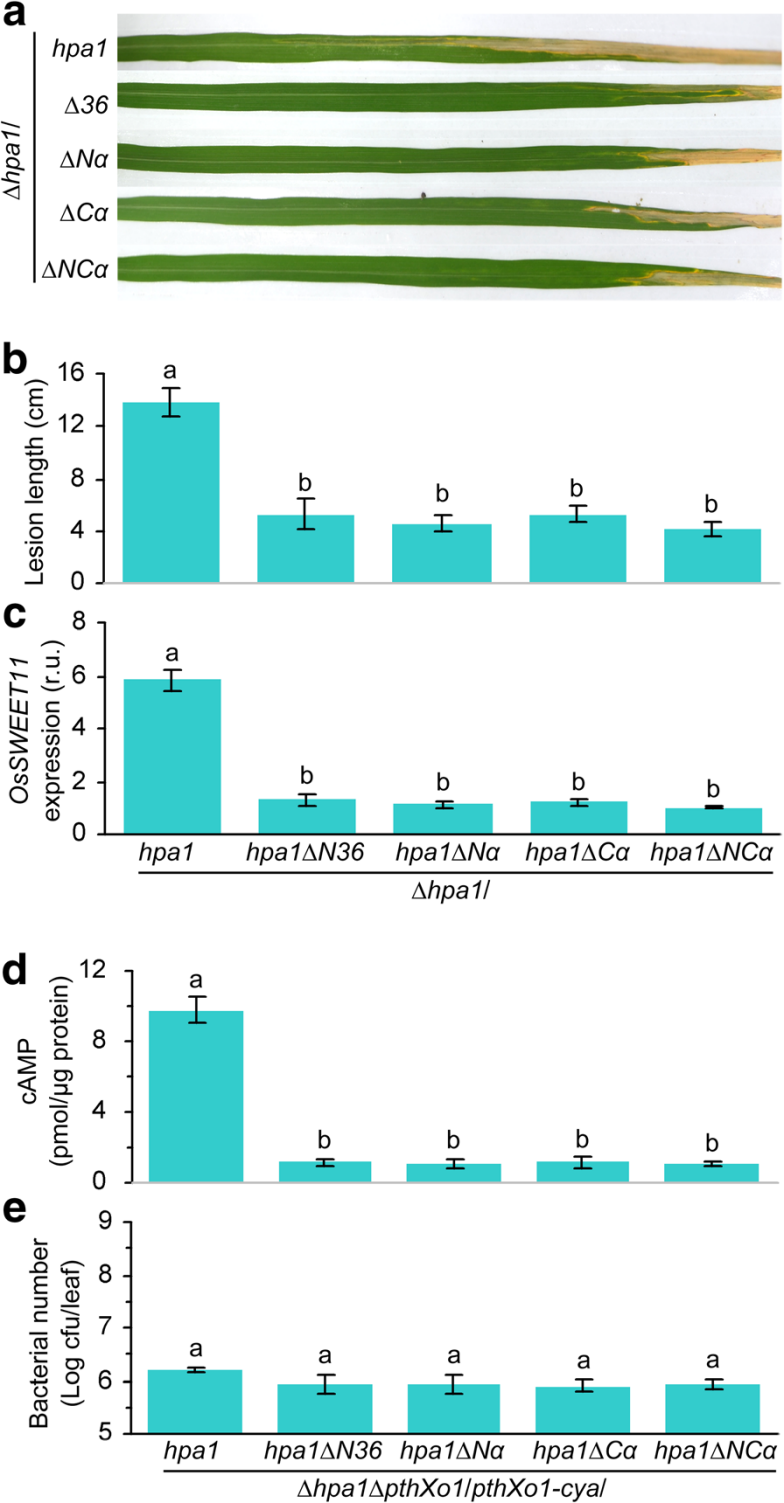
Hpa1 α-helices play a critical role in PthXo1 translocation. **a**–**e**, Bacterial *hpa1* mutants *∆N36*, *∆Nα*, *∆Cα*, and *αNC* were generated by deleting *hpa1* sequence regions, which encode the N-terminal region made of 36 residues, the N-terminal α-helix, the C-terminal α-helix, and both α-helices, respectively. Resulting mutant genes were introduced into the *∆hpa1* or *∆hpa1∆pthXo1/pthXo1-cya* mutant of *Xoo* strain PXO99. Recombinant bacteria were used in Nipponbare inoculation by the leaf-top-clipping method. Inoculated plants were subjected to the following analyses. (**a**) Bacterial blight symptoms on leaves photographed at day 12 after inoculations. **b** Blight lesion length on leaves from (**a**). **c** Relative units (r.u.) of *OsSWEET11* gene expression in leaves at 12 hpi. The average expression level of *OsSWEET11* in the plant with Δ*hpa1*/*hpa1ΔNC* α-helix was defined as 1 to evaluate relative extents of gene expression in plants inoculated with other *Xoo* strains. (**d**) The content of cAMP from PthXo1-Cya activity in cytoplasm of leaf cells at 12 hpSi. In (**b**) to (**e**), data are the means ± SEMs; different letters on bar graphs indicate significant differences in multiple comparisons for the bacterial strains; *P* < 0.05; *n* = 9 repetitions from 3 independent experiments each involving 3 repetitions

**Fig. 8:**
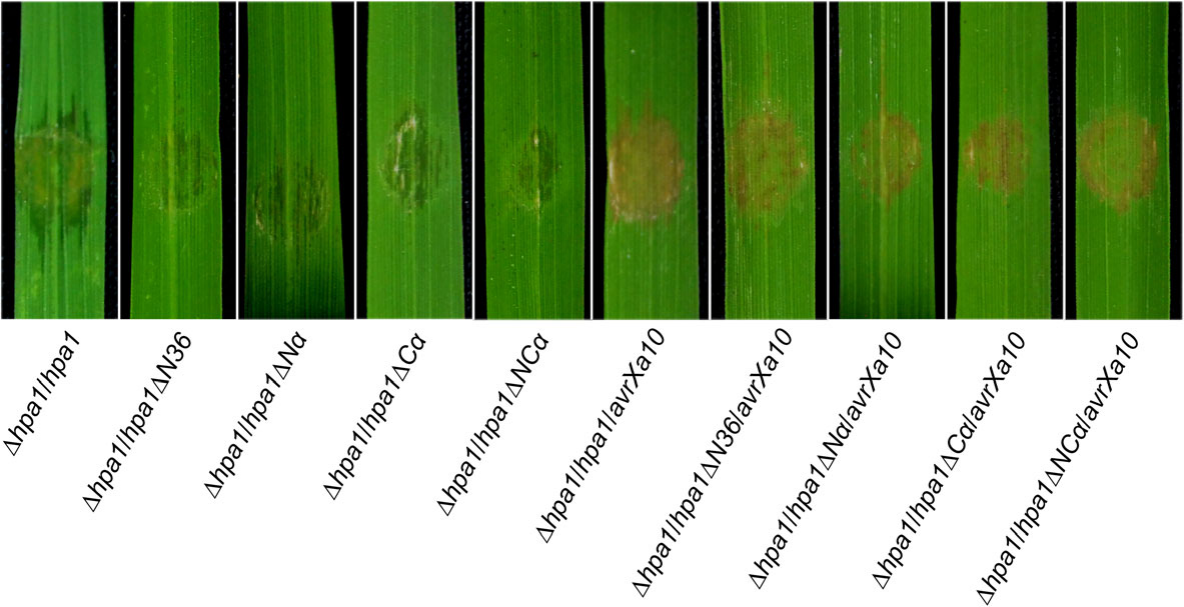
Deletions of *hpa1* sequence regions affect TAL effector functions. TAL effector functions refer to the virulent role of PthXo1 and the avirulent role of ArvXa10 on IRBB10. Fourteen-day-old IRBB10 seedlings were inoculated by leaf infiltration with every bacterial suspension of the indicated strains; leaves were photographed at 5 dpi

Given that the α-helix motif present in the N-terminal region of Hpa1 on its bioactivities [5, 14, 24, 25], we assumed that the motif is likely to serve as a determinant of the translocator function. This hypothesis was validated by fragment deletions toward the Hpa1 sequence (Fig. 6), followed by investigating the pathological performance of Hpa1 (Fig. 7). We generated Hpa1 mutant versions ∆N36, ∆Nα, ∆Cα, and ∆NCα by deleting the first 36 residues, N- terminal α-helix, C-terminal α-helix, and both α-helices, respectively (Fig. 6). All mutant versions attenuated the virulent role of PthXo1 (Fig. 7) and impaired the avirulent function of AvrXa10 (Fig. 8). Virulence impairment by Hpa1 mutations caused evident reductions in both severities of bacterial blight (Fig. 7a, b) and levels of *OsSWEET11* expression (Fig. 7c) in Nipponbare. In Cya reporting assays (Fig. 7c) with similar populations of different bacterial strains (Fig. 7e), PthXo1 translocation was severely compromised by Hpa1 mutations compared to that observed with the canonical protein (Fig. 7d).

## Discussion

We have identified Hpa1 as a translocator of TAL effectors, PthXo1 and AvrXa10 of *Xoo*, based on the quantitative changes in translocation and function of both TAL effectors with and without Hpa1. The performance of Hpa1 in all parallel experiments were in agreement with the functional characteristics of type III translocators. In essence, the translocators regulate the translocation of certain effectors, rather than the secretion by T3SS, and regulate virulent or avirulent roles of effectors depending on plant responses [8, 9, 22]. Therefore, we conclude that Hpa1 functions as a type III hydrophilic translocator that regulates the virulent role of PthXo1 and the avirulent role of AvrXa10 in susceptible and resistant rice varieties, respectively.

The translocator Hpa1 may not be specifically required for the translocation of selected T3SS substrates but also contributes to the translocation of all effectors of a given bacterial strain. This hypothesis remains to be validated in the future by studying more effectors, at least including Xops. Indeed, characterization of the molecular mechanism that governs effector translocation is merely in the initial stage while numerous studies are needed to elucidate translocation of different effectors from plant-pathogenic bacteria. For example, an *Xoo* strain produces near 30 Xops [18] but none of them have been studied with respect to translocation. Moreover, the composition of type III translocon and the number of translocators present in a species or a strain of plant-pathogenic bacteria are still not known although the issues have been well demonstrated in animal-pathogenic bacteria [5]. While many works are needed to identify the translocator repertoire and translocon composition, the characterization of Hpa1 as a TAL effector translocator is a definite step forward to better understanding of the composition of type III translocon in plant-pathogenic bacteria.

In addition to Hpa1, HrpF is also required for TAL effector translocation [22, 28]. HrpF is the first type III translocator discovered in plant-pathogenic bacteria and has been described as a translocator of AvrBs3 in *X. campestris* pv. *vesicatoria* [22], the bacterial spot pathogen of pepper [38]. HrpF is highly conserved in the *Xanthomonas* genus [36] and contains two predicted TM domains [22, 39], which are characteristic of type III hydrophobic translocators [2, 5]. The C-terminal region of HrpF is essential for AvrBs3 translocation, whereas the N-terminus contains a secretion signal. Thus, secretion and translocation are sequential but independent processes, in agreement with HcrV-dependent secretion and Hpa1-mediated translocation of *Xoo* PthXo1 and AvrXa10 (Fig. 2).

Additional translocators may cooperate with Hpa1 and HrpF to mediate TAL effector translocation. Sugio and colleagues (2005) found that AvrXa10-containing *hrpF hpa1* double mutant was impaired in virulence and avirulence to susceptible and resistant rice varieties IR24 and IRBB10, respectively. As the authors stated that the double mutant elicited the HR in IRBB10 and noted “data not shown”, we guess that the HR extent was probably weakened in line with the avirulence reduction by HrpF and Hpa1 knockout. As the extent of virulence or avirulence depends on the quantity of effector translocation, the performance of *hrpF hpa1* double mutant [36] is consistent with the effects of Hpa1 on PthXo1 and AvrXa10 translocation observed in our study. Sugio and colleagues (2005) hypothesized that effector translocation may involve additional translocators, plant cell endocytosis, direct secretion after pilus penetration of plant cells, or unknown effects of HrpF on effector activities. Animal-pathogenic bacteria possess one hydrophilic and two hydrophobic translocators, which are concomitantly required for effector translocation and are assumed to be assembled chronologically into a translocon [2, 5]. This model agrees not only with the performance of *hrpF hpa1* double mutant [36] but also with the critical role of Hpa1 in TAL effector translocation. Further evidence is challenging yet fairly worthy to elucidate whether effector translocation is subject to multiple regulations, including plant cell endocytosis, effector import into plant cells after pilus penetration of plant PMs, and the effect of HrpF or the other translocators on effector activities.

It was reported that *Xoo* HrpF needed Hpa2 to coregulate AvrXa10 translocation [28]. Hpa2 is a type III accessary protein and a lytic transglycosylase that decomposes bacterial cell walls [40]. In the canonical secretion of type III accessary proteins, lytic transglycosylases potentially associate with the bacterial periplasm and degrade the peptidoglycan substrate [41, 42]. This activity presumably enlarges the periplasmic mesh pores to accommodate the T3SS machinery, which then extend to the interface between bacterial and eukaryotic cells [43–45]. Subsequently, a hydrophilic translocator is secreted to initiate translocon assembly [2, 3, 6–9]. Therefore, Hpa1 may be critical for translocon formation as a prerequisite for TAL effector translocation [5].

The protein mutation analysis suggests that the pair of α-helix motif is important for the function of Hpa1 as a type III translocator. However, the present data can not exclude other parts of the Hpa1 sequence. All the truncated Hpa1 proteins tested in this study, including the version lacking the N-terminal 36 amino acid residues, showed loss of virulence/avirulence functions like the *hpa1* null mutant. Therefore, it is necessary to study in the future whether the biological function of Hpa1 involves other parts of the protein sequence.

## Conclusions

Meticulous genetic analyses indicate that Hpa1 contributes to a substantial part of the virulent role that PthXo1 plays in the susceptible rice variety. Genetic data also supports a marked effect of Hpa1 on the avirulent role of AvrXa10 in the resistant rice variety. Immunoblotting analysis demonstrates the critical function of Hpa1 in mediating the effector translocation from bacterial cells into cytosol of rice cells. This function of Hpa1 as a type III translocator has subsequent effects on the TAL effector target genes in terms of expression levels. This establishes the mechanistic linkage between Hpa1 recognition by rice plasma membrane and pathological role of the effectors that experience translocation under regulation by the translocator. We do not know how Hpa1 cooperates with HrpF in the hypothetic translocon assembly, and this will the subject of further studies.

## Additional file

Additional file 1: **Table S1.** Strains and plasmids used and created in this study. **Table S2.** Information on genes tested and primers used in this study. (DOCX 35 kb)

## Abbreviations

Cya: calmodulin-dependent adenylate cyclase
HR: hypersensitive response
PM: plasma membrane
T3SS: type III secretion system
TAL: transcription activator-like
*Xoo*: *Xanthomonas oryzae* pv. *oryzae*, pathogen of rice bacterial blight
Xop: *Xanthomonas* outer proteins
