# Supplementary material for: Hpa1 is a type III translocator in Xanthomonas oryzae pv. oryzae
Source: BMC Microbiol. 2018 Sep 4;18:105. doi: 10.1186/s12866-018-1251-3 (PMC6123991; doi:10.1186/s12866-018-1251-3)
Supplement: Supplementary file 1 — Table S1. Strains and plasmids used and created in this study. Table S2. Information on genes tested and primers used in this study. (DOCX 35 kb) [file 12866_2018_1251_MOESM1_ESM.docx]

**Supplemental Data**

**Additional file 1: Table S1.** Strains and plasmids used and created in this study

| Strains or Plasmids | Relevant characteristics | Source/Reference |
| --- | --- | --- |
| Strains  *Escherichia coli*  DH5α  BL21(DE3)  *X. oryzae* pv. *Oryzae*  PXO99  P*Δhpa1*  P*ΔpthXo1*  P*Δhpa1ΔpthXo1*  P*ΔhrcV*  P*Δhpa1/hpa1*  P*ΔpthXo1/pthXo1*  P*Δhpa1ΔpthXo1/hpa1*  */pthXo1*  PXO99/*avrXa10*  P*Δhpa1/avrXa10*  P*ΔpthXo1/avrXa10*  P*Δhpa1ΔpthXo1*  */avrXa10*  P*ΔhrcV/avrXa10*  P*ΔpthXo1/pthXo1-cya*  P*Δhpa1ΔpthXo1/pthXo1*  *-cya*  P*Δhpa1ΔpthXo1/hpa1*  */pthXo1-cya*  P*ΔhrcV/pthXo1-cya*  PXO99/*avrXa10-cya*  P*Δhpa1/avrXa10-cya*  P*ΔpthXo1/avrXa10-cya*  P*ΔhrcV/avrXa10-cya*  P*ΔpthXo1/pthXo1-blaM*  P*Δhpa1ΔpthXo1/pthXo1*  *-blaM*  P*ΔhrcV/pthXo1-blaM*  PXO99/*avrXa10-blaM*  P*Δhpa1/avrXa10-blaM*  P*ΔhrcV/avrXa10-blaM*  P*Δhpa1/hpa1ΔN36*  P*Δhpa1/hpa1ΔNα*  P*Δhpa1/hpa1ΔCα*  P*Δhpa1/hpa1ΔNCα*  P*Δhpa1ΔpthXo1/pthXo1*  *-cya/hpa1ΔN36*  P*Δhpa1ΔpthXo1/pthXo1*  *-cya/hpa1ΔNα*  P*Δhpa1ΔpthXo1/pthXo1*  *-cya/hpa1ΔCα*  P*Δhpa1ΔpthXo1/pthXo1*  *-cya/hpa1ΔNCα*  P*Δhpa1ΔpthXo1/pthXo1*  *-blaM /hpa1*  P*Δhpa1ΔpthXo1/pthXo1*  *-blaM /hpa1ΔN36*  P*Δhpa1ΔpthXo1/pthXo1*  *-blaM /hpa1ΔNα*  P*Δhpa1ΔpthXo1/pthXo1*  *-blaM /hpa1ΔCα*  P*Δhpa1ΔpthXo1/pthXo1*  *-blaM /hpa1ΔNCα*  Plasmids  pK18*sacB*  pHM1  pZW*pthXo1*  pZW*avrXa10*  pZW*pthXo1-cya*  pZW*avrXa10-cya*  pZW*pthXo1-blaM*  pZW*avrXa10-blaM*  pMD18-T simple  pMS107  pET30a (+)  pET41a (+) | F^–^ 80d*lacZ* M15(*lacZYA-argF*) U169 *endA1 deo*R *rec*A1 *hsd*R17(r_K_^–^ m_K_^+^) *pho*A *sup*E44 λ^–^ *thi-l gyr*A96 *rel*A1  F^–^ *omp*T *hsd*SB (*r_B_^–^ m_B_^–^*) *gal dcm* (DE3)  Philippine race 6; azacytidine resistant clone of PXO99,  virulent to rice cultivars Nipponbare and IRBB10  PXO99 *hpa1* unmarked mutant  PXO99 *pthXo1* unmarked mutant  PXO99 *hpa1pthXo1* double unmarked mutant  PXO99 *hrcV* knock-out mutant  PXO99 *hpa1* mutant complemented with pHM*hpa1*  PXO99 *pthXo1* mutant complemented with pHM*pthXo1*  PXO99 *hpa1pthXo1* mutant complemented with pHM*hpa1pthXo1*  PXO99 transformed with pHM*avrXa10*  PXO99 *hpa1* mutant transformed with pHM*avrXa10*  PXO99 *pthXo1* mutant transformed with pHM*avrXa10*  PXO99 *hpa1pthXo1* mutant transformed with pHM*avrXa10*  PXO99 *hrcV* mutant transformed with pHM*avrXa10*  PXO99 *pthXo1* mutant complemented with pHM*pthXo1-cya*  PXO99 *hpa1pthXo1* mutant complemented with pHM*pthXo1-cya*  PXO99 *hpa1pthXo1* mutant complemented with pHM*hpa1pthXo1-cya*  PXO99 *hrcV* mutant transformed with pHM*pthXo1-cya*  PXO99 transformed with pHM*avrXa10-cya*  PXO99 *hpa1* mutant transformed with pHM*avrXa10-cya*  PXO99 *pthXo1* mutant transformed with pHM*avrXa10-cya*  PXO99 *hrcV* mutant transformed with pHM*avrXa10-cya*  PXO99 *pthXo1* mutant complemented with pHM*pthXo1-blaM*  PXO99 *hpa1 pthXo1* mutant complemented with pHM*pthXo1-blaM*  PXO99 *ΔhrcV* mutant transformed with pHM*pthXo1-blaM*  PXO99 transformed with pHM*avrXa10-blaM*  PXO99 *hpa1* mutant transformed with pHM*avrXa10-blaM*  PXO99 *hrcV* mutant transformed with pHM*avrXa10-blaM*  PXO99 *hpa1* mutant complemented with pHM*hpa1ΔN36 (hpa1* sequence with a deletion of 1-36 sites in N-terminal)  PXO99 *hpa1* mutant complemented with pHM*hpa1ΔNα (hpa1* sequence with a deletion of α-helix in N-terminal)  PXO99 *hpa1* mutant complemented with pHM*hpa1ΔCα (hpa1* sequence with a deletion of α-helix in C-terminal)  PXO99 *hpa1* mutant complemented with pHM*hpa1ΔNCα (hpa1* sequence with two deletions of α-helices in NC-terminals)  PXO99 *hpa1 pthXo1* mutant complemented with pHM*hpa1ΔN36-pthXo1-cya*  PXO99 *hpa1 pthXo1* mutant complemented with pHM*hpa1ΔNα-pthXo1-cya*  PXO99 *hpa1 pthXo1* mutant complemented with pHM*hpa1ΔCα-pthXo1-cya*  PXO99 *hpa1 pthXo1* mutant complemented with pHM*hpa1ΔNCα-pthXo1-cya*  PXO99 *hpa1 pthXo1* mutant complemented with  pHM*hpa1-pthXo1-blaM*  PXO99 *hpa1 pthXo1* mutant complemented with pHM*hpa1ΔN36-pthXo1-blaM*  PXO99 *hpa1 pthXo1* mutant complemented with pHM*hpa1ΔNα-pthXo1-blaM*  PXO99 *hpa1 pthXo1* mutant complemented with pHM*hpa1ΔCα-pthXo1-blaM*  PXO99 *hpa1 pthXo1* mutant complemented with pHM*hpa1ΔNCα-pthXo1-blaM*  Suicide vector derivative from pK18mobGII, *sacB^+^*, Km^R^  Broad-host range vector with pUC19 polylinker, Sp^R^  PthXo1 fused to *lacZ* promoter of pBluescript II KS(+)  AvrXa10 fused to *lacZ* promoter of pBluescript II KS(+)  Cya tag inserted in the *Sac* I site of pZW*pthXo1*  Cya tag inserted in the *Sac* I site of pZW*avrXa10*  BlaM tag inserted in the *Sal* I site of pZW*pthXo1*  BlaM tag inserted in the *Sal* I site of pZW*avrXa10*  pUC *ori*, cloning vector, Amp^R^  pLC20H containing the PCR fragment of *cyaA* from nucleotide 4 to nucleotide 1221, Amp^R^  pBR322 origin, *T7* promoter His-tag, Km^R^  pBR322 origin, *T7* promoter His-tag, GST-tag, Km^R^ | This lab  Novagen  This lab  This lab  This study  This study  This study  This lab  This study  This study  This study  This study  This study  This study  This study  This study  This study  This study  This study  This study  This study  This study  This study  This study  This study  This study  This study  This study  This study  This study  This study  This study  This study  This study  This study  This study  This study  This study  This study  This study  This study  This study  This study  This lab  This lab  Yang et al., 2006  Makino et al., 2006  This study  This study  This study  This study  Takara  This lab  Novagen  Novagen |

**Additional file 2: Table S2.** Information on genes tested and primers used in this study

|  | Primers / product size (bp) / subjects |
| --- | --- |
| *hpa1* (ACD56757) | 5’-ATGAATTCTTTGAACACACAAT-3’, 5’-TTACTGCATCGATGCGCTGTCG-3’ / 420 / coding sequence cloning by PCR |
|  | 5’-TTCTCAACAACGCCCCGCGGATTTG -3’,  5’-TTACTGCATCGATGCGCTGTCG-3’ / 639 / promoter + coding sequence |
|  | Upstream homologous arm:  5’-CG**GGATCC**CGCTGCTGCAAATAGAATACG-3’ (*Bam*H I),  5’-GTAGGGGCGACCAACAGTTCTCGTGACGATTCCTCTCTGATT-3’ / 537,  Downstream homologous arm:  5’-AATCAGAGAGGAATCGTCACGAGAACTGTTGGTCGCCCCTAC-3’,  5’-GC**TCTAGA**TCACTCGCACATGCTGGTTCT-3’ (*Xba* I) / 536 / deletion |
|  | 5’-AA**CTGCAG**TTCTCAACAACGCCCCGCGGATTTG-3’ (*Pst* I),  5’-GG**GGTACC**TTActtatcgtcgtcatccttgtaatc(flag)CTGCATCGATGCGCTGTCGCT-3’ (*Kpn* I) / 639 / complemention |
|  | 5’-acaagtttgtacaaaaaagcaggctctccaaccacc(homologous arm)ATGAATTCTTTGAACACACAAT-3’,  5’-tccgccaccaccaaccactttgtacaagaaagctgggta(homologous arm)CTGCATCGATGCGCTGTCGC-3’ / 417 / SUB Y2H |
|  | 5’-CGG**GGTACC**ATGAATTCTTTGAACACACAAT-3’ (*Kpn* I),  5’-CGC**GGATCC**CTGCATCGATGCGCTGTCG-3’ (*Bam*H I) / 417 / BiFC |
|  | 5’-CGG**GGTACC**ATGAATTCTTTGAACACACAAT-3’ (*Kpn* I),  5’-CGC**GGATCC**TTActtatcgtcgtcatccttgtaatc(flag)CTGCATCGATGCGCTGTCG-3’ (*Bam*H I) / 420 / Co-IP |
|  | 5’-CGC**GGATCC**ATGAATTCTTTGAACACACAAT-3’ (*Bam*H I),  5’-CCC**AAGCTT**TTACTGCATCGATGCGCTGTCG-3’ (*Hin*d III) / 420 / GST-Pulldown. |
| *hpa1ΔN(1-36)* | 5’-TTCTCAACAACGCCCCGCGGATTTG -3’,  5’-ACTGGTCCAGTTGCTTTTCCATCGTGACGATTCCTCTCTGATTA-3’ / 219 / promoter  5’-TAATCAGAGAGGAATCGTCACGATGGAAAAGCAACTGGACCAGT-3’,  5’-CTGCATCGATGCGCTGTCGCT-3’ / 309 / *ΔN(1-36)* coding sequence / recombination |
|  | 5’-AA**CTGCAG**TTCTCAACAACGCCCCGCGGATTTG-3’ (*Pst* I),  5’-GG**GGTACC**TTActtatcgtcgtcatccttgtaatc(flag)CTGCATCGATGCGCTGTCGCT-3’ (*Kpn* I) / 531 / complemention |
|  | 5’-acaagtttgtacaaaaaagcaggctctccaaccacc(homologous arm)ATGGAAAAGCAACTGGACCAGT-3’,  5’-tccgccaccaccaaccactttgtacaagaaagctgggta(homologous arm)CTGCATCGATGCGCTGTCGC-3’ / 309 / SUB Y2H |
|  | 5’-CGG**GGTACC**ATGGAAAAGCAACTGGACCAGTTGC-3’ (*Kpn* I),  5’-CGC**GGATCC**CTGCATCGATGCGCTGTCG-3’ (*Bam*H I) / 309 / BiFC |
|  | 5’-CGG**GGTACC**ATGGAAAAGCAACTGGACCAGTTGC-3’ (*Kpn* I),  5’CGC**GGATCC**TTACTTATCGTCGTCATCCTTGTAATCCTGCATCGATGCGCTGTCG-3’ (*Bam*H I) / 312 / Co-IP |
|  | 5’-CGC**GGATCC**ATGGAAAAGCAACTGGACCAGTTGC-3’ (*Bam*H I),  5’-CCC**AAGCTT**TTACTGCATCGATGCGCTGTCG-3’ (*Hin*d III) / 312 / GST-Pulldown |
| *hpa1ΔNɑ-helix* | 5’-TTCTCAACAACGCCCCGCGGATTTG -3’,  5’-CCTCAGCATTTTTGCTCGACTGCGAGATGCCCTGGTTGCCGC-3’ / 327 / promoter + coding sequence (1-108)  5’-GCGGCAACCAGGGCATCTCGCAGTCGAGCAAAAATGCTGAGG-3’,  5’-CTGCATCGATGCGCTGTCGCT-3’ / 261 / coding sequence (157-417) / recombination |
| *hpa1ΔCɑ-helix* | 5’-TTCTCAACAACGCCCCGCGGATTTG-3’,  5’-CACCAGCACCACCACCATTCTGCGAGGGGCCATTCTGCTGCCCA-3’ / 477 / promoter + coding sequence (1-258)  5’-TGGGCAGCAGAATGGCCCCTCGCAGAATGGTGGTGGTGCTGGTG-3’,  5’-CTGCATCGATGCGCTGTCGCT-3’ / 111 / coding sequence (307-417) / recombination |
| *hpa1ΔNCɑ-helices* | 5’-TTCTCAACAACGCCCCGCGGATTTG -3’,  5’-CCTCAGCATTTTTGCTCGACTGCGAGATGCCCTGGTTGCCGC-3’ / 327 promoter + coding sequence (1-108)  5’-GCGGCAACCAGGGCATCTCGCAGTCGAGCAAAAATGCTGAGG-3’,  5’-CACCAGCACCACCACCATTCTGCGAGGGGCCATTCTGCTGCCCA-3’ / 102 / coding sequence (157-258)  5’-TGGGCAGCAGAATGGCCCCTCGCAGAATGGTGGTGGTGCTGGTG-3’,  5’-CTGCATCGATGCGCTGTCGCT-3’ / 111 / coding sequence (307-417) / recombination |
| *pthXo1* (AY495676) | Upstream homologous arm:  5’-CG**GGATCC**CTATTCGGATTTCGTGCTGTAC-3’ (*Bam*H I),  5’-CAAACTGCTATGCCCAGGCTTATCGTTGTGCTCGTCCCTTTTG-3’ / 513  Downstream homologous arm:  5’-CAAAAGGGACGAGCACAACGATAAGCCTGGGCATAGCAGTTTG-3’,  5’-GC**TCTAGA**ATCGTGGAATTCGCCAAGGAAT-3’ (*Xba* I) / 605 / deletion |
| *avrXa10*  (U50552)  *cya* (Y00545)  *blaM*(JX268626) | 5’-GC**GAGCTC**gcatggttgatggagctattgcctcagtcaggctcagtcggagggacgatc(*TAL*)  GGGCAGCAATCGCATCAGGCT(*cya*)-3’ (*Sac* I),  5’-GC**GAGCTCAAGCTT**TTAGCTGTCATAGCCGGAATCC-3’ (*Sac* I-*Hin*d III) / 1221 / complemention, secretion and translocation |
|  | 5’-ACGC**GTCGAC**CCATGAGTATTCAACATTTCCGTG-3’ (*Sal* I),  5’-ACGC**GTCGAC**GCCAATGCTTAATCAGTGAGG-3’ (*Sal* I) / 861 / complemention and translocation |
| *SWEET11* (AK070510) | 5’-TGGTTCTGCTACGGCCTCTT-3’,  5’-GGTACCAGAAGTAGAGCCCCATCT-3’ / 103 / real-time RT-PCR |
| *Xa10* (ABA94452) | 5’-GGCATCATCTTCTCCGGCG-3’,  5’-GCAGCTATACGGGCATAAG-3’ / real-time RT-PCR |
| *Ubi1*  (U37687) | 5’-CCAGTAAGTCCTCAGCCATG-3’, 5’-TTTCAGACACCATCAAACCAG-3’ / 173 / real-time RT-PCR |
